# Rhabdomyosarcoma: A Case Report and Comprehensive Literature Review

**DOI:** 10.7759/cureus.88028

**Published:** 2025-07-15

**Authors:** Sebastián Dufner Krieger, Roberto A Hidalgo Ramos, Daniela Secades, Isaac Hong, Marcelo Ortiz

**Affiliations:** 1 General Medicine, University of Costa Rica, San José, CRI; 2 Medicine, University of Costa Rica, San José, CRI

**Keywords:** case report, chemotherapy, embryonal rhabdomyosarcoma, ophthalmic neoplasm, orbital mass, orbital tumor, pediatric oncology, radiotherapy, rhabdomyosarcoma, soft tissue sarcoma

## Abstract

Rhabdomyosarcoma (RMS) is an uncommon and aggressive malignancy originating from mesenchymal tissue, frequently affecting children. Its clinical presentation can vary significantly depending on the tumor's location and histological subtype, often complicating timely diagnosis. Early recognition and the initiation of appropriate multimodal treatment are essential to improving outcomes. This report describes a case of embryonal RMS (ERMS) in a young infant with a rapidly enlarging orbital mass and provides an overview of relevant diagnostic considerations and treatment strategies. The case highlights the value of early detection and coordinated, multidisciplinary care in achieving the best possible prognosis.

## Introduction

Orbital rhabdomyosarcoma (RMS) is the most common primary malignant orbital tumor of childhood, accounting for approximately 10% of all RMS cases and up to 90% of primary orbital malignancies in pediatric populations [1,2]. The estimated global incidence is four to five cases per million children under the age of 20 annually, with a peak occurrence between the ages of seven and eight years [3]. Approximately 90% of orbital RMS cases are diagnosed before age 16, although rare cases have been reported in both infants and adults [4,5]. A slight male predominance has been observed in several large case series [6].

Histologically, RMS is classified into four major subtypes: embryonal, alveolar, pleomorphic, and spindle cell/sclerosing. Among these, the embryonal subtype is most frequently encountered in orbital presentations, accounting for more than 90% of cases in children and associated with a more favorable prognosis [7].

Clinically, orbital RMS typically presents with rapidly progressive unilateral proptosis, often accompanied by eyelid edema, ptosis, conjunctival chemosis, pain, and, in some cases, visual impairment due to optic nerve compression [8]. The tumor most often arises from the superonasal quadrant of the orbit and may secondarily involve surrounding structures, including the extraocular muscles, optic nerve, eyelid, conjunctiva, and, more rarely, the uveal tract [9]. The clinical presentation can closely resemble other orbital conditions, such as cellulitis or idiopathic inflammation, which can delay diagnosis.

If untreated, RMS has a high propensity for local invasion, including erosion of orbital bones and intracranial extension via the superior orbital fissure or optic canal. Although distant metastases are relatively uncommon at initial diagnosis, hematogenous dissemination to the lungs, bone marrow, and skeletal system may occur, particularly in advanced or relapsed cases [10].

Historically, the prognosis for orbital RMS was poor, with three-year survival rates below 30% when orbital exenteration was the standard therapeutic approach [11]. Recognizing the need for less mutilating and more effective alternatives, the Intergroup Rhabdomyosarcoma Study Group (IRSG) was established in 1972 under the US National Cancer Institute to develop and evaluate multimodal treatment strategies. Through a series of landmark clinical trials, the IRSG demonstrated that a combined approach involving chemotherapy, radiotherapy, and selective conservative surgery significantly improved survival [12]. Five-year survival rates now exceed 90% in cases of localized embryonal orbital RMS [13].

Diagnosis involves a combination of imaging, typically MRI for soft tissue detail and CT for bone involvement, and histopathological confirmation via biopsy. Immunohistochemical markers such as desmin, myogenin, and MyoD1 aid in differentiating RMS from other small round blue cell tumors of childhood [14]. A complete metastatic workup, including chest CT, bone scan, and bone marrow biopsy, is also standard.

Treatment is tailored based on clinical risk group, tumor location, and histologic subtype. Current protocols such as those of the Children’s Oncology Group (COG) typically employ vincristine, actinomycin D, and cyclophosphamide (VAC) chemotherapy, along with precise radiotherapy targeting orbital structures. Surgical intervention is limited to cases with residual or refractory disease [15].

This article presents a focused review of pediatric orbital RMS, including clinical presentation, diagnostic strategy, treatment evolution, and current evidence-based management protocols.

## Case presentation

**Figure 1:**
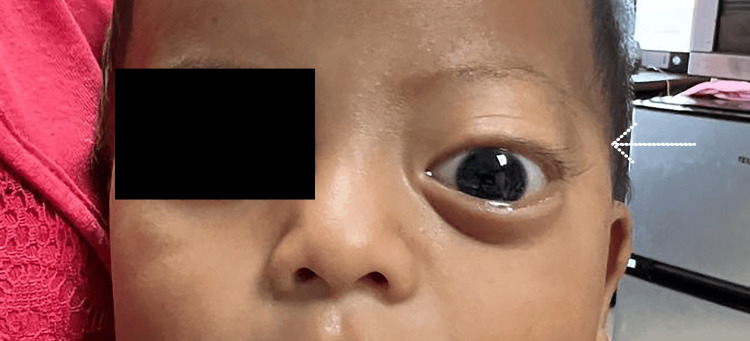
Left orbit demonstrating a soft-tissue mass causing proptosis and inferior displacement of the left globe in the child with orbital rhabdomyosarcoma. The white arrow highlights the mass. Written informed consent to include the image in an open-access article was obtained from the patient's parent/guardian.

A seven-month and 28-day-old male infant from the rural Caribbean town of Sixaola, Costa Rica, was brought to the emergency department of Tony Facio Hospital by his parents due to a two-week history of progressive left-eye proptosis. The parents denied fever, trauma, vomiting, visual disturbances, seizures, or other systemic symptoms. The proptosis had an insidious onset but had noticeably worsened over the previous week, prompting medical evaluation (Figure 1).

On physical examination, the infant was alert, afebrile, and hemodynamically stable. Notable findings included marked proptosis of the left eye with mild inferior displacement of the globe. There was no conjunctival injection, chemosis, or ocular discharge. The globe was intact, and extraocular motility was restricted in upward gaze. There was no afferent pupillary defect. Examination of the right eye and the rest of the systemic and neurologic examination was unremarkable.

Initial laboratory workup, including a complete blood count, C-reactive protein, and basic metabolic panel, revealed no abnormalities. The patient presented with progressive left eye proptosis, discomfort, and mild visual disturbances, raising concern for an underlying orbital process. Microbiological cultures were not obtained, as the clinical presentation lacked signs suggestive of orbital or periorbital cellulitis, such as fever, leukocytosis, erythema, or purulent discharge, and no infectious process was suspected.

Regarding tumor marker profiles, it is important to clarify that RMS does not typically produce specific serum tumor markers that are useful for diagnosis or monitoring. Common markers such as alpha-fetoprotein (AFP), beta-human chorionic gonadotropin (β-hCG), lactate dehydrogenase (LDH), and neuron-specific enolase (NSE) are either not elevated in RMS or are nonspecific and not routinely used in clinical practice. As such, no tumor markers were indicated or obtained in this case. Diagnosis and risk stratification rely primarily on histopathology, immunohistochemistry, and imaging rather than on serum biomarkers.

Due to the high clinical suspicion of a space-occupying lesion, the patient was urgently referred to the National Children’s Hospital of Costa Rica (Hospital Nacional de Niños (HNN)) for further evaluation.

Upon admission to the pediatric oncology service, a multidisciplinary team consisting of pediatric oncology, ophthalmology, and pathology was assembled. A detailed ophthalmologic examination confirmed restricted extraocular movements and mild optic disc edema in the affected eye. No palliative measures were undertaken for the reduction of optic disc edema, as the degree of edema was mild and not associated with visual impairment or signs of elevated intracranial pressure; therefore, intervention was not considered necessary. The clinical picture raised strong suspicion for a primary orbital neoplasm, and an incisional biopsy was promptly scheduled.

**Figure 2:**
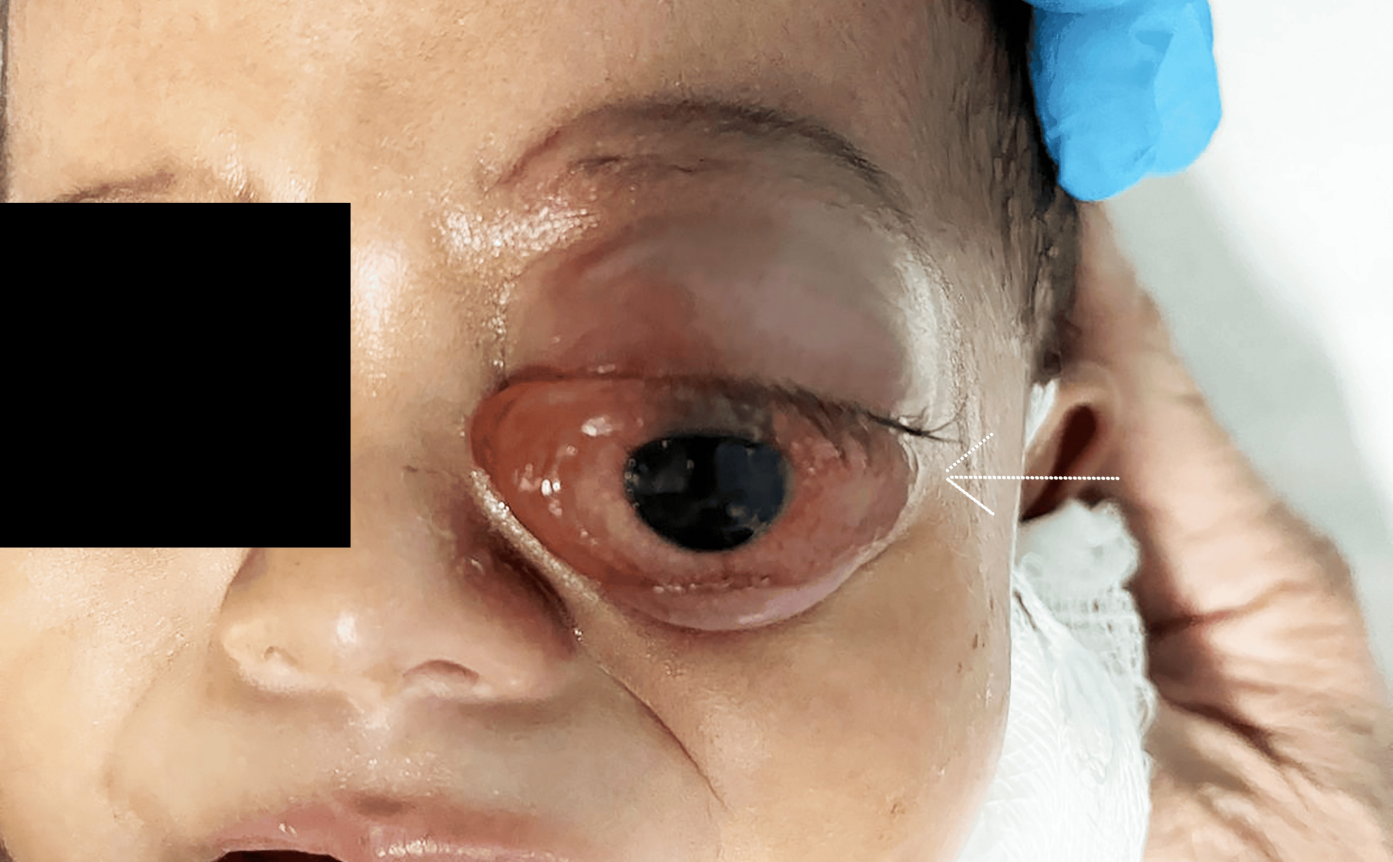
External photograph showing marked proptosis of the left eye with inferior displacement of the globe, one week after symptom onset in the child with orbital rhabdomyosarcoma. The white arrow indicates the direction of globe displacement. Written informed consent to include the image in an open-access article was obtained from the patient's parent/guardian.

During hospitalization, the patient’s clinical course deteriorated. Within one week, proptosis progressed significantly, and ocular motility became increasingly restricted (Figure 2).

**Figure 3:**
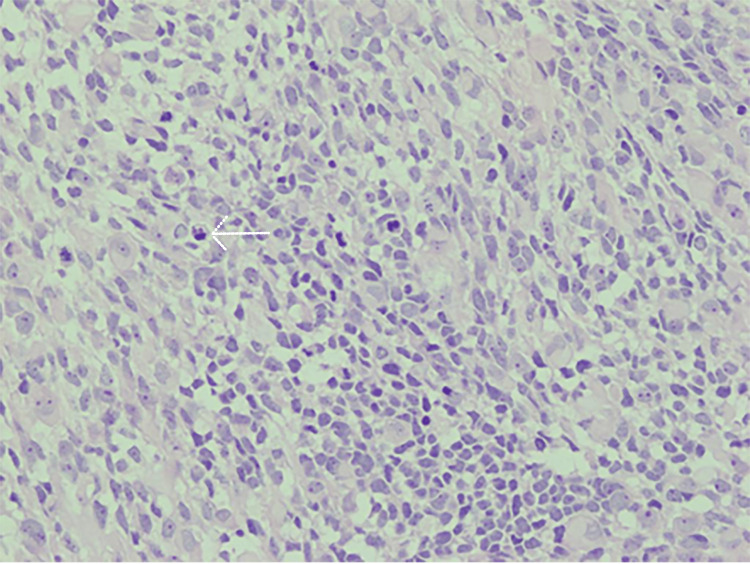
Histopathological image (H&E stain, ×40) showing sheets of small, round blue cells with scant eosinophilic cytoplasm, round nuclei with fine chromatin, and occasional mitotic figures. Spindle-shaped cells with rhabdomyoblastic differentiation are also visible. The white arrow highlights a rhabdomyoblast with eosinophilic cytoplasm and an elongated nucleus, characteristic of embryonal rhabdomyosarcoma.

The child was taken to the operating room for a surgical incisional biopsy of the posterosuperior orbital mass. Histopathological evaluation revealed a highly cellular malignant neoplasm, consistent with embryonal RMS (ERMS). Microscopic analysis demonstrated sheets of small, round blue cells with scant eosinophilic cytoplasm, round nuclei with fine chromatin, and occasional mitotic figures. Additionally, spindle-shaped cells with eosinophilic cytoplasm and features of rhabdomyoblastic differentiation were identified, further supporting the diagnosis (Figure 3).

Immunohistochemical staining demonstrated strong positivity for desmin, myogenin, and MyoD1, confirming the diagnosis of ERMS. Tumor cells were negative for lymphoid and neural markers, including CD45 and NSE, respectively.

CT imaging and genetic testing were not performed due to resource limitations at the time of evaluation. The diagnosis was established based on histopathological and immunohistochemical findings.

**Figure 4:**
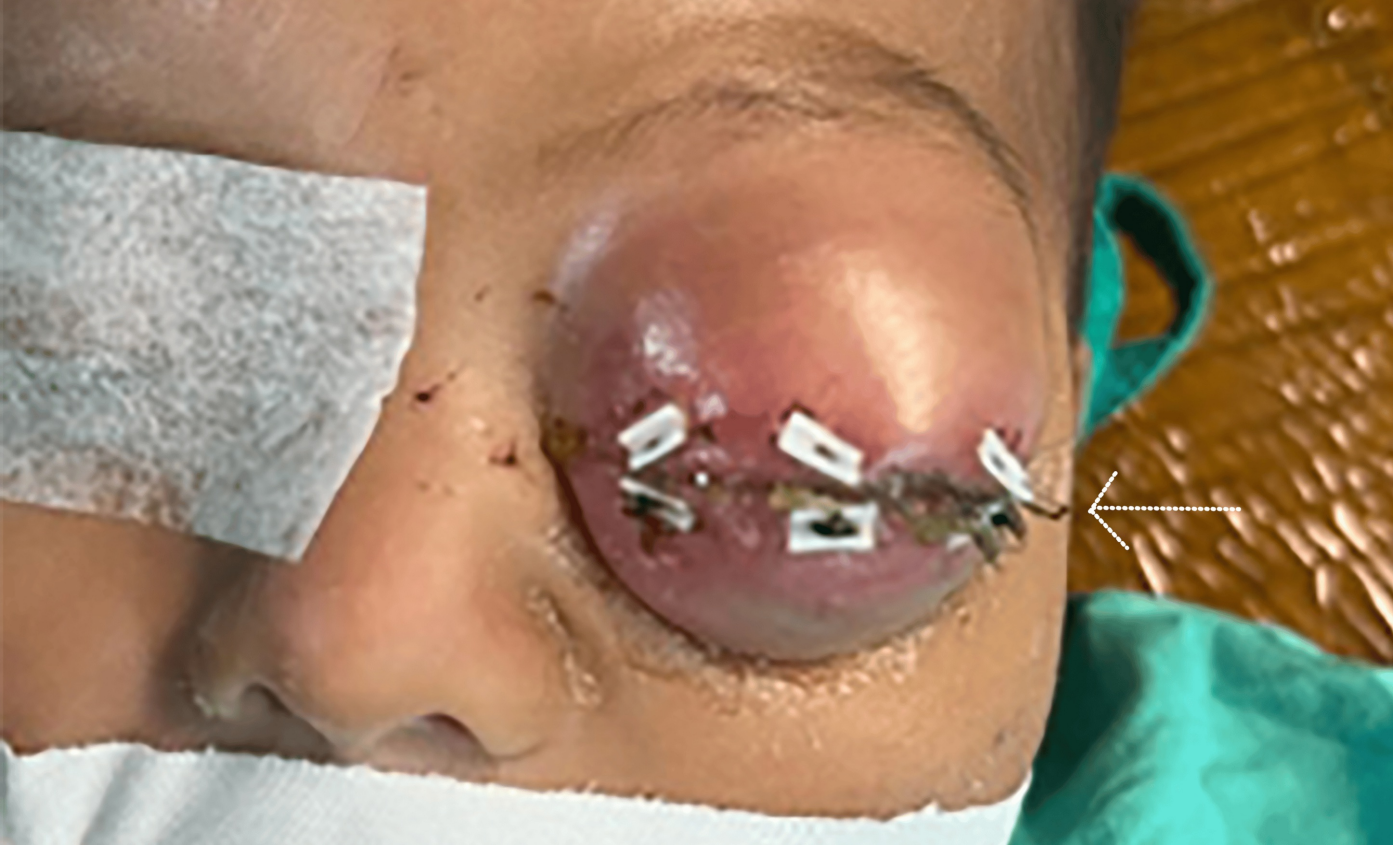
Postoperative external photograph showing the protective blepharoplasty performed to address progressive proptosis. The procedure involved narrowing of the palpebral fissure to reduce ocular surface exposure, preserve corneal integrity, and maintain globe retention within the orbit. The white arrow indicates the surgical margin of the blepharoplasty site. Written informed consent to include the image in an open-access article was obtained from the patient's parent/guardian.

Following confirmation of the diagnosis via histopathology and immunohistochemistry, the patient was classified as having group III orbital ERMS as per IRSG criteria. Treatment was initiated under the national pediatric oncology protocol, adapted from the COG guidelines. A VAC chemotherapy regimen, comprising vincristine, actinomycin D (dactinomycin), and cyclophosphamide, was started with dosing adjusted for the patient’s age and weight. Given the patient’s young age, chemotherapy was administered cautiously to minimize the risk of cumulative toxicity, consistent with pediatric dosing recommendations. The clinical plan included the use of radiotherapy as part of a combined modality approach; however, no further treatment details regarding timing, dose, or delivery method were available at the time of manuscript preparation. MRI and CT imaging were not performed, limiting anatomical mapping for radiotherapy; therefore, radiation planning was expected to rely on clinical and surgical findings. Advanced modalities such as proton beam therapy were not available in our setting, and conventional photon-based conformal radiotherapy was anticipated. Despite the prompt initiation of multimodal therapy, including systemic chemotherapy and localized radiotherapy in accordance with standard pediatric oncology protocols, the patient’s clinical condition deteriorated. Within one more week, the proptosis worsened markedly, compromising the integrity of the ocular surface. Due to the rapidly expanding nature of the tumor, the ophthalmology team performed a protective blepharoplasty to maintain globe retention within the orbit, minimize evaporative stress, and preserve corneal integrity. The procedure involved narrowing the palpebral fissure to reduce ocular surface exposure and delay corneal decompensation (Figure 4). 

**Figure 5:**
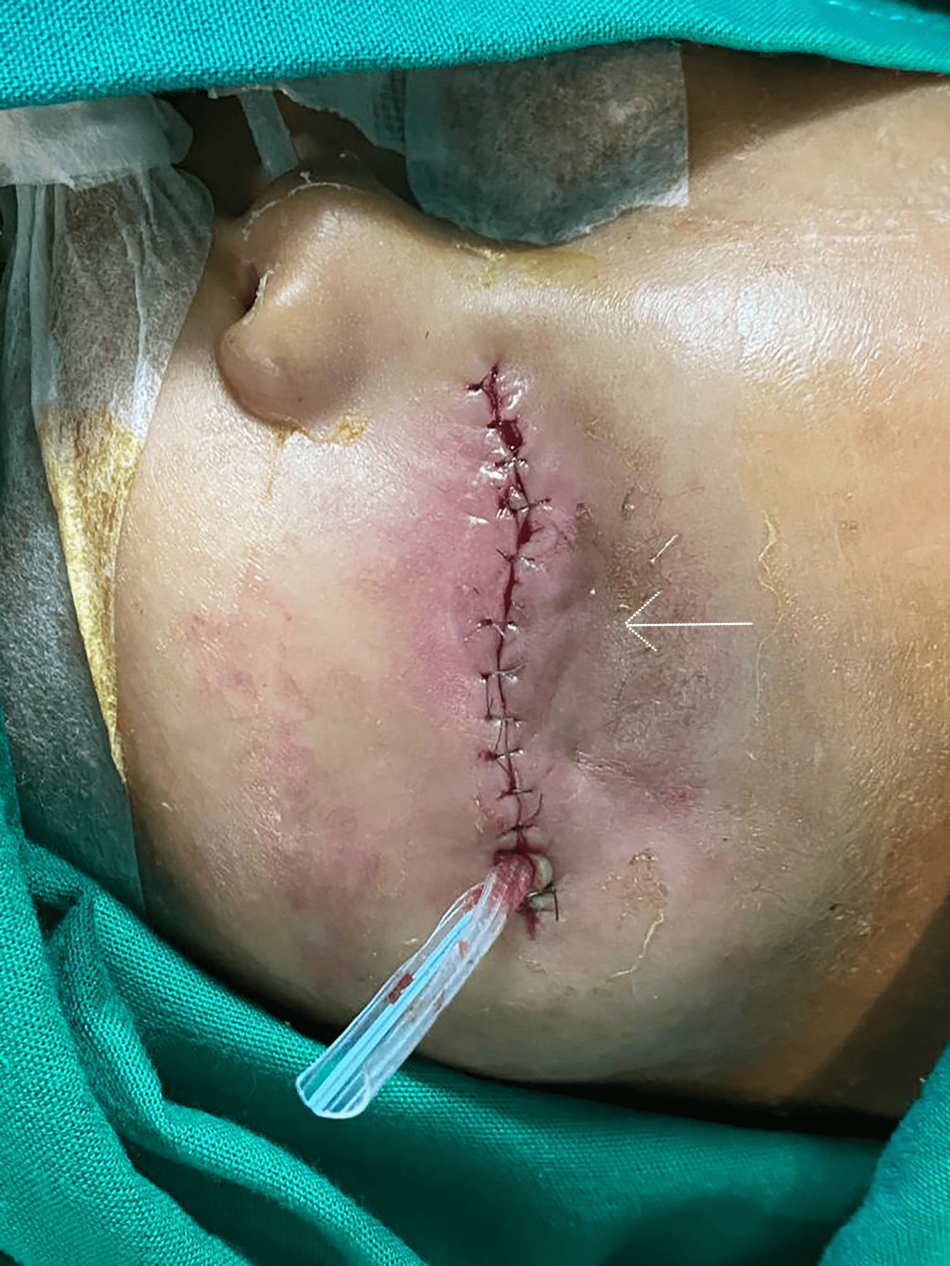
Postoperative image following left orbital exenteration. The procedure was performed to achieve local disease control after the tumor showed aggressive progression and poor response to initial therapies. The white arrow indicates the anatomical margin of the exenterated orbital cavity. Written informed consent to include the image in an open-access article was obtained from the patient's parent/guardian.

Nevertheless, the tumor continued to grow aggressively, and the patient’s response to initial treatments was suboptimal. After multidisciplinary deliberation involving pediatric oncology, ophthalmology, pediatric surgery, and ethics consultants, it was concluded that orbital exenteration was warranted. The decision prioritized the patient’s long-term survival and quality of life, as local disease control was deemed unachievable with conservative measures alone. Exenteration was performed successfully, and the postoperative course was uneventful. The patient was subsequently maintained on adjunct chemotherapy and enrolled in a close follow-up program that included periodic surveillance imaging to monitor for local recurrence or distant metastasis (Figure 5). At the time of manuscript submission, no additional clinical updates were available regarding long-term outcome or disease progression.

## Discussion

RMS may arise in any anatomical location containing skeletal muscle, as well as in sites where skeletal muscle is not typically present, such as the urinary bladder, common bile duct, and the soft tissues of the orbit [8]. Anatomically, RMS most commonly involves the head and neck region (40%), followed by the genitourinary tract (20%), extremities (20%), trunk (10%), and other sites (10%) [8]. Approximately 25% of RMS cases involving the head and neck region originate in the orbit [9].

Due to its orbital location, the most frequent presenting sign of orbital RMS is unilateral proptosis, often accompanied by slight inferior displacement of the globe [10]. The acute onset and rapidly progressive nature of the condition may mimic infectious or inflammatory etiologies [8]. Occasionally, a palpable mass or bluish-purple discoloration beneath the eyelid may be observed, along with other clinical findings such as conjunctival injection, chemosis, ptosis, eyelid swelling, and facial asymmetry [10,11]. Posteriorly located tumors may present with choroidal folds, ophthalmoplegia, or optic nerve compression. In rare cases, the orbit may be secondarily involved by metastases from distant primary tumors or by direct extension from the paranasal sinuses or nasopharynx [9].

A definitive diagnosis of orbital RMS requires tissue biopsy. Depending on tumor location and suspected dissemination, an incisional or excisional biopsy is preferred. Fine needle aspiration (FNA) is discouraged, as it often yields insufficient material for immunohistochemistry or molecular studies [12,13].

From a histopathologic standpoint, RMS cells resemble immature skeletal muscle. Five main subtypes are recognized: embryonal, alveolar, pleomorphic, spindle cell, and botryoid [14]. Microscopically, the tumor typically shows round or spindle-shaped rhabdomyoblastic cells arranged in a loose syncytial pattern with evidence of skeletal muscle differentiation [15]. Among these subtypes, ERMS is the most common and is characterized by round, oval, elongated, or stellate nuclei and abundant eosinophilic cytoplasm [15].

Genetic profiling further supports subtype classification. ERMS is frequently associated with loss of heterozygosity at chromosome 11p15.5. In contrast, the alveolar subtype often presents with chromosomal translocations such as t(2;13)(q35;q14) or t(1;13)(p36;q14), resulting in PAX3/7-FOXO1 fusion genes. These fusions encode aberrant transcription factors that inhibit myogenic differentiation and promote tumor proliferation [11].

In evaluating rapidly progressive proptosis in children, the differential diagnosis should include both benign and malignant entities. These encompass orbital cellulitis, idiopathic orbital inflammatory syndrome (orbital pseudotumor), granulocytic sarcoma, non-Hodgkin lymphoma, metastatic neuroblastoma, and Langerhans cell histiocytosis, among others [12]. Clinical overlap with infectious processes is common, and RMS is frequently misdiagnosed as orbital cellulitis. However, the absence of systemic signs such as fever or lethargy, along with poor response to antibiotics, should prompt consideration of a malignant process [15]. Supportive laboratory tests, including complete blood count, erythrocyte sedimentation rate, and C-reactive protein, may aid in differentiating inflammatory from neoplastic causes.

A summary of selected reported cases of orbital ERMS, including presentation, treatment, and outcomes, is presented in Table 1.

**Table 1:** Summary of published cases of orbital embryonal rhabdomyosarcoma in pediatric patients

Study (Author, Year)	Patient Age and Sex	Tumor Location	Histologic Subtype	Presenting Symptoms	Imaging Findings	Treatment Administered	Clinical Outcome
Siktberg et al., 2022 [3]	2-year-old female	Orbit	Embryonal	Persistent orbital mass following treatment	CT showed residual soft-tissue thickening	Surgical excision followed by systemic chemotherapy and radiotherapy	Histologically inactive residual tissue; no active tumor detected
Shields et al., 2001 [8]	Mean: 7.5 years (range not reported)	Orbit	Embryonal (most common subtype)	Proptosis, eyelid swelling, ptosis	CT and MRI showed a mass displacing the globe	Surgical biopsy, radiotherapy, and systemic chemotherapy	Five-year overall survival greater than 90% for low-risk cases
Yazıcı et al., 2014 [10]	9-year-old male	Orbit	Embryonal	Eyelid mass, progressive proptosis	CT revealed a well-defined intraorbital lesion	Surgical resection followed by systemic chemotherapy	No recurrence after three years of follow-up
Present case	7-month-old male	Left orbit	Embryonal	Rapid-onset proptosis, restricted upward gaze	Clinical examination suggested a posterosuperior orbital mass with optic nerve compression	Incisional biopsy, systemic chemotherapy, protective blepharoplasty, and orbital exenteration	Alive and stable; ongoing chemotherapy and clinical monitoring

Recent studies show that approximately 20% of newly diagnosed patients with orbital ERMS and up to 50% of those with recurrent disease undergo orbital exenteration [16,17]. These findings emphasize the critical role that radiotherapy and high-intensity chemotherapy have come to play in achieving local control and long-term survival [18,19].

Treatment protocols differ between regions. European approaches typically aim to reduce long-term radiation-related toxicity, while North American strategies prioritize minimizing recurrence through early use of radiotherapy [19]. Systemic chemotherapy remains the cornerstone of treatment, aiming to shrink the primary tumor and address microscopic or metastatic disease. Local control is pursued via surgery, radiotherapy, or both. Complete resection is often unfeasible due to anatomical constraints or the desire to preserve function and appearance.

Radiotherapy is typically reserved for residual or unresectable tumors and is adjusted according to risk stratification. In North America, the most commonly used chemotherapy regimen is VAC. In Europe, ifosfamide is frequently substituted for cyclophosphamide. A randomized clinical trial comparing these regimens found no significant difference in overall survival [16-18].

While high-dose chemotherapy and stem cell rescue have been explored for metastatic disease, evidence suggests no meaningful survival advantage and a higher risk of treatment-related adverse events [20]. Likewise, although doxorubicin is widely used in soft tissue sarcomas, its benefit in RMS remains uncertain.

Risk stratification, based on tumor size, nodal involvement, histologic subtype, metastatic status, and surgical margins, plays a central role in guiding treatment intensity [16-20]. Radiation dosing varies accordingly: 36 Gray for positive microscopic margins, 41.4 Gray for nodal involvement, and up to 50.4 Gray for gross residual disease.

Despite its effectiveness, radiotherapy is associated with long-term complications, including radiation-induced dermatitis, bone growth disturbances, and secondary malignancies. Therefore, minimizing toxicity is essential in pediatric patients requiring long-term follow-up [16-20].

Recent genomic and preclinical research has dramatically advanced our understanding of RMS biology, emphasizing molecular stratification and targeted interventions. Alveolar RMS (ARMS) is defined by PAX3/7-FOXO1 fusion proteins, present in approximately 60-80% of cases. These fusion oncoproteins act as master regulators of tumorigenesis via transcriptional reprogramming and super-enhancer activation and are associated with poor prognosis and aggressive phenotypes [21,22]. In contrast, ERMS, which lacks these fusions, frequently harbors alternate molecular alterations such as RAS pathway (HRAS/KRAS/NRAS) and TP53 mutations, suggesting distinct therapeutic vulnerabilities [22].

Targeted therapies are emerging from these molecular insights. In particular, KDM family epigenetic inhibitors, such as P3FI‑90, have demonstrated potent anti-tumor activity in preclinical fusion-positive RMS models by deactivating PAX-FOXO1-mediated transcription [21]. Another promising strategy involves co-inhibition of mTOR and IGF-1R pathways. A phase I study combining temsirolimus (mTOR inhibitor) with cixutumumab (IGF-1R antibody) showed tolerability and early efficacy signals in pediatric sarcomas, including RMS [23].

For RAS-mutant ERMS, MEK inhibitors such as trametinib have shown efficacy in suppressing oncogenic signaling and promoting differentiation in preclinical models [24]. In addition, BRD4 inhibitors, such as JQ1, have demonstrated success in targeting MYC-regulated super-enhancer networks in RMS, thereby disrupting transcriptional drivers of tumor proliferation [25].

Although these therapies are not yet standard of care, they represent the future of precision oncology in pediatric RMS, particularly in relapsed or high-risk cases. In our case, which involved orbital ERMS, treatment followed standard protocol with VAC chemotherapy and radiotherapy; however, as molecular profiling becomes more widely accessible, it will play an essential role in guiding patient selection for targeted trials and individualized therapies.

This case underscores the importance of early recognition and a coordinated multidisciplinary approach in managing orbital ERMS. While most cases respond to standard therapy, aggressive tumors may require escalation to surgical interventions such as blepharoplasty or exenteration. A thorough understanding of the clinical spectrum and treatment variability is essential for optimizing outcomes.

## Conclusions

Orbital ERMS should be considered in the differential diagnosis of pediatric patients presenting with acute-onset proptosis or atypical orbital masses. Early recognition and prompt initiation of multimodal therapy are critical to improving outcomes. Current evidence supports combined chemoradiation, using VAC chemotherapy and tailored radiotherapy, as the preferred first-line approach for intermediate-risk, group III disease. Surgical intervention is generally reserved for cases with poor response or progressive disease.

Although advanced imaging and molecular testing were not available in this case, their role is increasingly important in guiding risk stratification and identifying candidates for emerging targeted therapies. This case highlights the need for a multidisciplinary approach, especially in resource-limited settings, to balance oncologic control with functional preservation and quality of life.
